# Supplementary material for: Isolation and evolutionary analyses of porcine epidemic diarrhea virus in Asia
Source: PeerJ. 2020 Oct 20;8:e10114. doi: 10.7717/peerj.10114 (PMC7583610; doi:10.7717/peerj.10114)
Supplement: Supplemental Information 1 [file peerj-08-10114-s001.pdf]

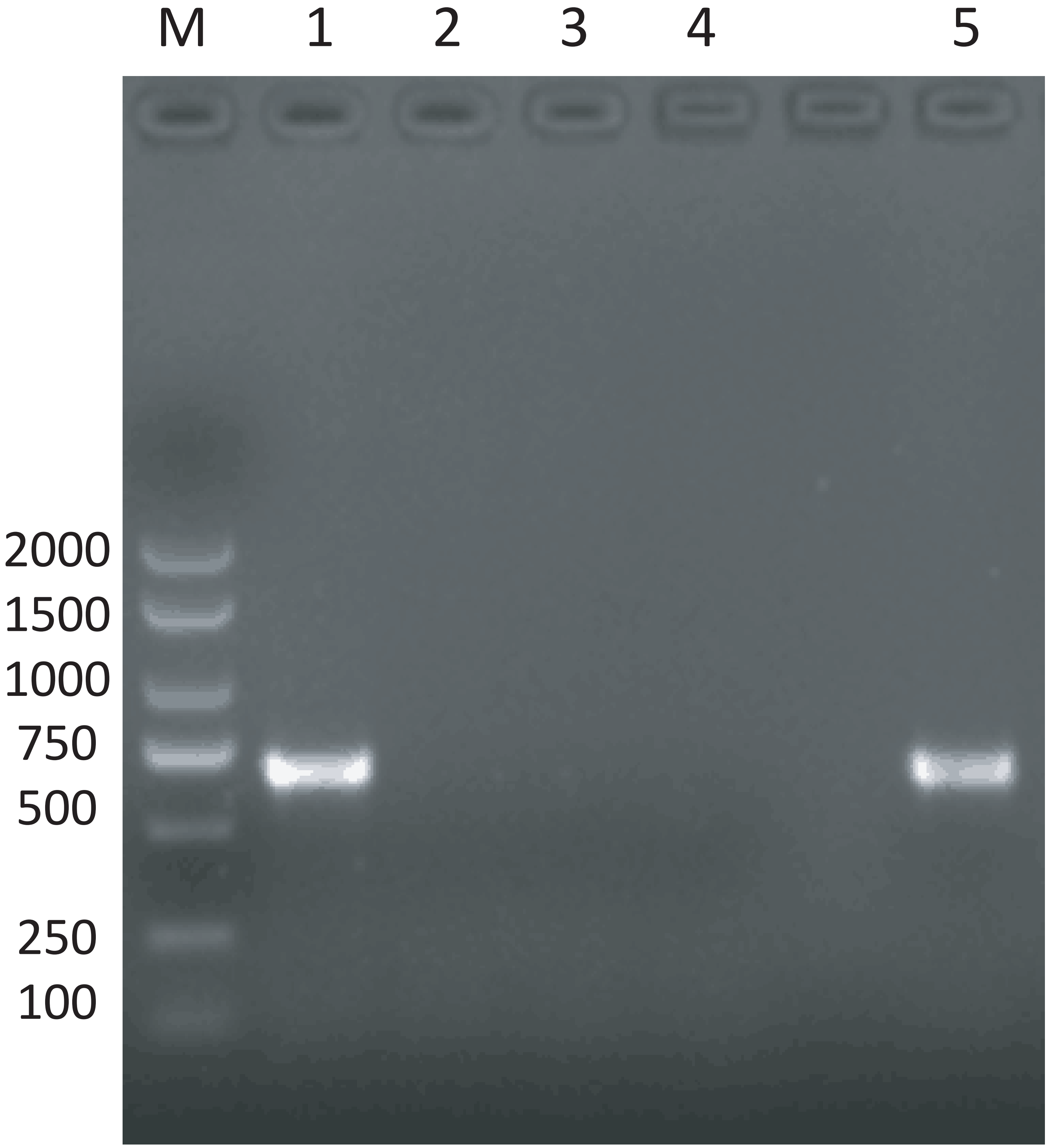

M: DL2000 marker
1. Sample detected for PEDV
2. Sample detected for TGEV
3. Sample detected for PoRV
4. Negative control
5. Positive control
