# Supplementary figures and images for: Isolation and evolutionary analyses of porcine epidemic diarrhea virus in Asia
Source: PeerJ. 2020 Oct 20;8:e10114. doi: 10.7717/peerj.10114 (PMC7583610; doi:10.7717/peerj.10114)

**A.**

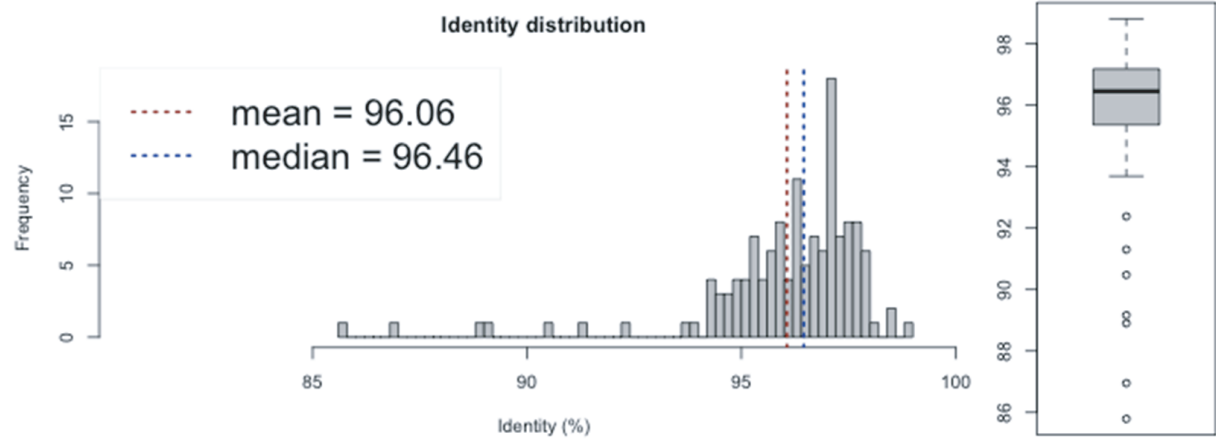

**B.**

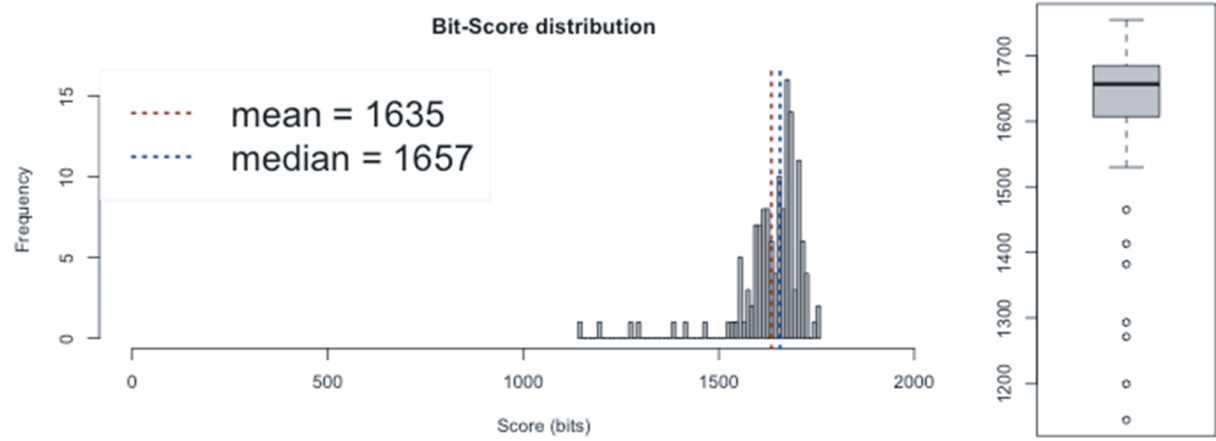

Supplement: Supplemental Information 2 [file peerj-08-10114-s002.pdf]
