# Supplementary material for: Isolation and evolutionary analyses of porcine epidemic diarrhea virus in Asia
Source: PeerJ. 2020 Oct 20;8:e10114. doi: 10.7717/peerj.10114 (PMC7583610; doi:10.7717/peerj.10114)
Supplement: Supplemental Information 3 — The red box indicates the amino acids which are the same between CV777 and HB2018. [file peerj-08-10114-s003.pdf]

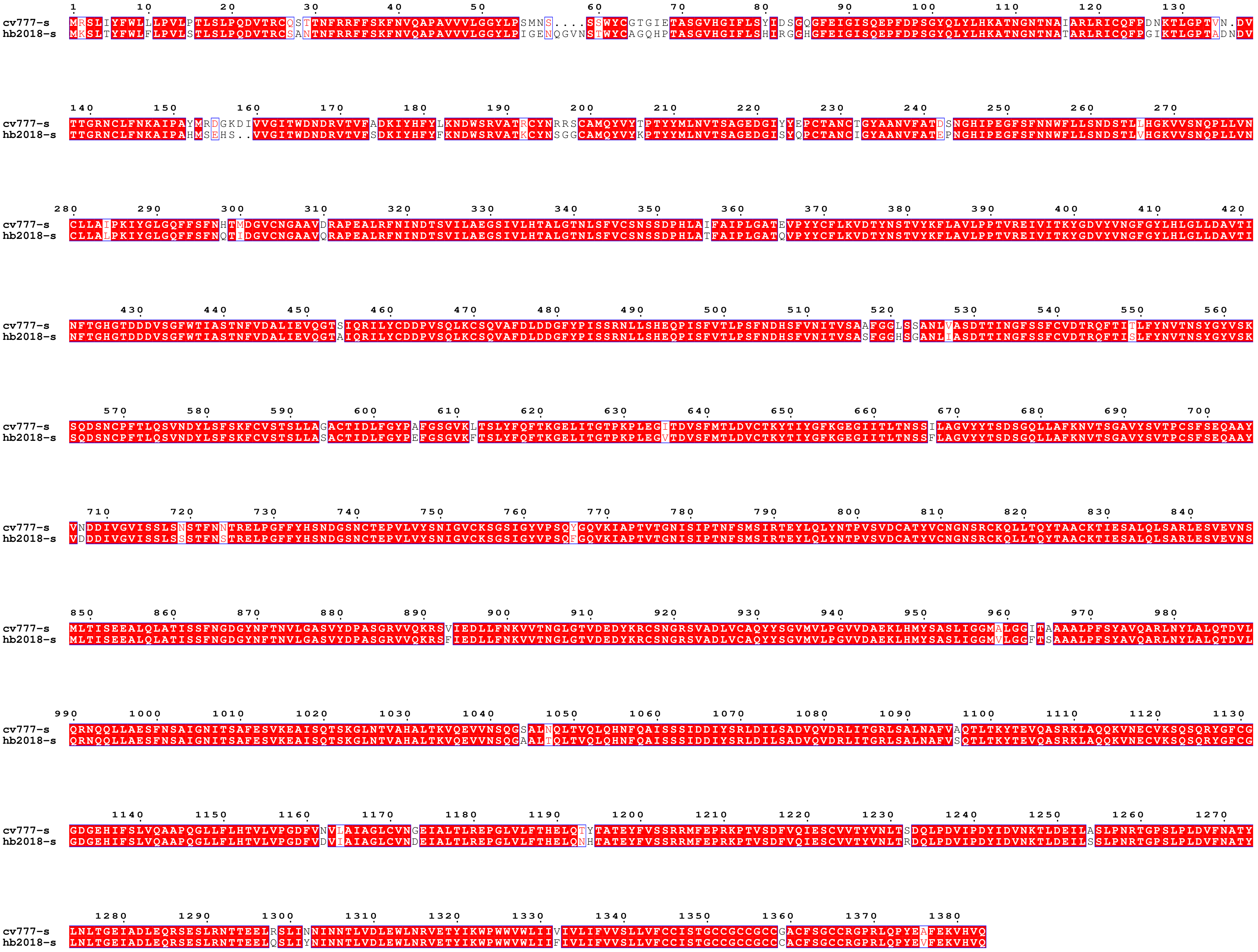
cv777-s
hb2018-s
