# Supplementary material for: Isolation and evolutionary analyses of porcine epidemic diarrhea virus in Asia
Source: PeerJ. 2020 Oct 20;8:e10114. doi: 10.7717/peerj.10114 (PMC7583610; doi:10.7717/peerj.10114)
Supplement: Supplemental Information 5 [file peerj-08-10114-s005.docx]

**Table S2 Amino acid changes, deletions, and/or insertions in the S protein of HB2018 compared to that of CV777**

|  | **2** | **5** | **10** | **15** | **27** | **28** | **29** | **55** | **56** | **57** | **58** | **59** | **60** | **61** | **63** | **65** | **69** | **70** | **72** | **73** |
| --- | --- | --- | --- | --- | --- | --- | --- | --- | --- | --- | --- | --- | --- | --- | --- | --- | --- | --- | --- | --- |
| **CV777** | R | I | L | P | Q | S | T | - | - | - | - | - | S | M | S | S | G | A | I | E |
| **HB2018** | K | T | F | S | S | A | N | I | G | E | N | Q | G | V | - | T | - | T | Q | H |
|  | **86** | **88** | **89** | **91** | **122** | **132** | **133** | **140** | **159** | **161** | **162** | **163** | **164** | **165** | **166** | **181** | **189** | **199** | **203** | **204** |
| **CV777** | Y | D | S | Q | I | D | N | V | Y | R | D | G | K | D | I | A | L | R | R | R |
| **HB2018** | H | R | G | H | T | G | I | A | H | S | E | H | S | - | - | S | F | K | S | G |
|  | **205** | **213** | **230** | **232** | **239** | **249** | **250** | **273** | **291** | **305** | **307** | **372** | **461** | **524** | **528** | **530** | **534** | **556** | **601** | **612** |
| **CV777** | S | T | Y | E | T | D | S | L | I | H | M | E | S | A | L | S | V | T | G | A |
| **HB2018** | G | K | S | Q | I | E | P | V | L | Q | I | Q | A | S | H | G | I | S | S | E |
|  | **619** | **642** | **674** | **714** | **726** | **731** | **773** | **900** | **966** | **970** | **972** | **1051** | **1054** | **1103** | **1169** | **1171** | **1180** | **1200** | **1201** | **1239** |
| **CV777** | L | I | I | N | N | N | Y | V | A | I | A | S | N | A | N | L | G | T | Y | S |
| **HB2018** | F | V | F | D | S | S | F | F | V | F | S | A | T | S | D | I | D | N | H | R |
|  | **1261** | **1305** | **1309** | **1339** | **1366** | **1383** |  |  |  |  |  |  |  |  |  |  |  |  |  |  |
| **CV777** | A | R | N | V | G | A |  |  |  |  |  |  |  |  |  |  |  |  |  |  |
| **HB2018** | S | Q | Y | F | C | V |  |  |  |  |  |  |  |  |  |  |  |  |  |  |
