# Supplementary material for: Isolation and evolutionary analyses of porcine epidemic diarrhea virus in Asia
Source: PeerJ. 2020 Oct 20;8:e10114. doi: 10.7717/peerj.10114 (PMC7583610; doi:10.7717/peerj.10114)
Supplement: Supplemental Information 6 [file peerj-08-10114-s006.docx]

**Table S3 Amino acid changes, deletions, and/or insertions in the S proteins of the S proteins of the Chinese GI-b strains compared to those of the Chinese GI-a strains**

|  | **2** | **3** | **15** | **74** | **122** | **161** | **191** | **292** | **320** | **334** | **364** | **368** | **373** | **388** | **531** | **647** | **778** | **780** |
| --- | --- | --- | --- | --- | --- | --- | --- | --- | --- | --- | --- | --- | --- | --- | --- | --- | --- | --- |
| **GI-a strains** | K/R | S | S/P | A | N | V | L | L | N/D | S | S/L | A | A | R | S/L | E | P | Y |
| **GI-b strains** | T | P | L | D | S | - | I | W | Q | F | K | T | V | S | H | Q | L | D |
|  | **820** | **891** | **905** | **977** | **1037** | **1061** | **1078** | **1181** | **1188** | **1274** | **1348** |  |  |  |  |  |  |  |
| **GI-a strains** | A | A | R | V | K | N | L | A | E | N | - |  |  |  |  |  |  |  |
| **GI-b strains** | V | V | S | L | N | T | I | D | D | I | V |  |  |  |  |  |  |  |
