# Supplementary material for: Isolation and evolutionary analyses of porcine epidemic diarrhea virus in Asia
Source: PeerJ. 2020 Oct 20;8:e10114. doi: 10.7717/peerj.10114 (PMC7583610; doi:10.7717/peerj.10114)
Supplement: Supplemental Information 7 [file peerj-08-10114-s007.docx]

**Table S4 Amino acid changes, deletions, and/or insertions in the S proteins of the Chinese GII strains compared to those of the Chinese GI strains**

|  | **27** | **28** | **29** | **55** | **56** | **57** | **58** | **59** | **60** | **61** | **62** | **64** | **68** | **69** | **70** | **71** | **72** | **82** |
| --- | --- | --- | --- | --- | --- | --- | --- | --- | --- | --- | --- | --- | --- | --- | --- | --- | --- | --- |
| **GI strains** | Q | S | T | - | - | - | - | S | M | N | S | S | G | T | G | I | E | L |
| **GII strains** | S | A | N | I | G | E | N | Q | G | V | N | T | A | G | Q | H | P | V |
|  | **84** | **86** | **87** | **89** | **124** | **134** | **135** | **142** | **144** | **161** | **162** | **163** | **164** | **165** | **166** | **167** | **168** | **183** |
| **GI strains** | Y | D | S | Q | I | D | N | V | - | Y | M | R/Q | D | G | K | N | D/I | A |
| **GII strains** | H | R | G | H | T | S | I | A | N | H | M | S | E | H | S | - | - | S |
|  | **188** | **191** | **201** | **205** | **206** | **207** | **215** | **232** | **234** | **1246** | **1312** | **1345** | **1346** | **1347** | **1348** |  |  |  |
| **GI strains** | H | L/I | R | K | R | S | T | Y | E | S | R | I | F | I | V |  |  |  |
| **GII strains** | Y | F | K | S | G | G | E | S | Q | R | Q | - | - | - | - |  |  |  |
